# Antibiotic Usage Prior and During Hospitalization for Clinical Severe Pneumonia in Children under Five Years of Age in Rabat, Morocco

**DOI:** 10.3390/antibiotics2040450

**Published:** 2013-09-26

**Authors:** Imane Jroundi, Rachid Benmessaoud, Chafiq Mahraoui, Cinta Moraleda, Houssain Tligui, Myriam Seffar, Badr Sououd Benjelloun, Jordi Vila, Joaquim Ruiz, Pedro L. Alonso, Quique Bassat

**Affiliations:** 1 Barcelona Centre for International Health Research (CRESIB), Rosselló 132, Barcelona E-08036, Spain; E-Mails: imane.jroundi@cresib.cat (I.J.); rachid.benmessaoud@cresib.cat (R.B.); cinta.moraleda@cresib.cat (C.M.); JVILA@clinic.ub.es (J.V.); JORUIZ@clinic.ub.es (J.R.); pedro.alonso@isglobal.org (P.L.A.); 2 École Nationale de Santé Publique (ENSP), Ministère de la santé, Rue Lamfadel Ach. Cherkaoui, Madinat Al Irfane, Rabat 6329, Morocco; 3 Hôpital d’Enfants (HER), Centre Hospitalier Universitaire Ibn Sina, Boulevard Ibn Rochd, Souissi, Rabat 10100, Morocco; E-Mails: cmahraoui@gmail.com (C.M.); tliguicom@yahoo.fr (H.T.); myriamseffar@yahoo.fr (M.S.); bsououd@gmail.com (B.E.B.)

**Keywords:** acute respiratory infection, antibiotics, children, risk factors, Morocco

## Abstract

Scarce and limited epidemiological, clinical and microbiological data are available regarding pediatric respiratory tract infections in the Kingdom of Morocco, a middle-income country in Northwestern Africa. Data on antibiotic usage for such infections are also scarce. A good understanding of pre-admission and intra-hospital usage of antibiotics in children with respiratory infections linked with an adequate surveillance of the antibiotic susceptibility from circulating pathogens could help policy makers improve their recommendations on management of respiratory infections. We hereby present data on antibiotic usage prior and during admission and antibiotic susceptibility of major circulating respiratory pathogens in children under five years of age admitted to the *Hôpital d’Enfants de Rabat*, Morocco, with a diagnosis of clinical severe pneumonia (using World Health Organization (WHO) standardized case definitions) during a period of 14 months (November 2010–December 2011), as part of a larger hospital-based surveillance study designed to understand the etiology and epidemiology of severe pneumonia cases among children.

## 1. Introduction

Acute respiratory infections (ARI) remain the leading cause of death in young children in low and middle income countries, accounting for almost 1.4 million annual deaths and 18% of the global deaths in children under five worldwide [1]. Recent estimates suggest that the global incidence of hospital admissions in young children related to severe or very severe lower respiratory tract infections may be as high as 11.9 million (95% CI 10.3–13.9 million) and 3.0 million (2.1–4.2 million) annual episodes respectively [2], representing a huge burden for the health systems, typically fragile in such settings.

ARI are among the most common causes of physician consultation in the pediatric age group [3]. Typically, the underlying etiology is viral, hence, not requiring—in principle—antibiotic prescription. At the outpatient level, however, antibiotics are often used for the treatment of many ARI episodes including unspecific symptoms such as sore throat, common cold and rhinitis or bronchospasm [4,5,6], for which there is an unlikely therapeutic benefit [7,8]. In other instances, the existing symptomatology, highly suggestive of a bacterial origin or with signs of severity, or a pre-existing co-morbidity predisposing to bacterial infections, clearly justifies the use of antibacterial agents. It has been estimated that 20% to 30% of all antimicrobial use is inappropriate [9], even if physicians have at their disposal World Health Organization (WHO) or Centers for Disease Control and Prevention (CDC)-proposed guidelines, algorithms and tools to help prevent misdiagnosis and inappropriate antibiotic usage [10,11].

The misuse of antibiotics can also lead to unnecessary side effects [12], such as rash, diarrhea, and rarely allergic reactions; pseudomembranous colitis secondary to *Clostridium difficile*; or more importantly, to the emergence of antibiotic resistance [13,14]. Factors leading to the misuse of antibiotics in children are complex, as cited in the literature, including individual physician prescribing practices [15], availability of over-the-counter drugs [16], or caretaker’s pressure, expectations, knowledge or beliefs [16,17,18,19,20]. 

Scarce and limited epidemiological, clinical and microbiological data are available regarding pediatric respiratory tract infections in the Kingdom of Morocco, a middle-income country in Northwestern Africa. The Ministry of Health has invested in the past decade in the reinforcement of several pneumonia control measures, including the strengthening of the Integrated Management of Childhood Illness (IMCI) program in remote areas [21], and more recently the introduction of conjugate vaccines against *Haemophilus influenzae* type b (2006) [22] and *Streptococcus pneumoniae* (13-valent, 2010). Possibly in relation to this, the proportion of total pediatric deaths attributable to pneumonia is believed to have decreased from 18% to 15% in the period of 2000 to 2012 [23], but the major gaps in knowledge regarding the precise etiology and epidemiology of pneumonia in Morocco remain inadequately addressed. Data on antibiotic susceptibility in Morocco are limited and disaggregated, although have suggested increasing trends of resistance or even multiresistance from *S. pneumoniae* isolates to penicillin or other commonly used antibiotics [24,25,26]. Data on antibiotic usage for respiratory infections are also scarce, although a recent survey conducted by the public health department reports that 56.5% of the approximately 130,000 children seen in primary health care settings with respiratory tract infections received antibiotics [27]. As antibiotics are available for purchase over-the-counter and patients do not require systematically a prescription from the physician to obtain these drugs, it is likely that real antibiotic usage is even higher. 

We hereby present data on antibiotic usage prior and during admission and antibiotic susceptibility of major circulating respiratory pathogens in children under five years of age admitted to the *Hôpital d’Enfants de Rabat*, Morocco, with a diagnosis of clinical severe pneumonia (using WHO standardized case definitions [28,29]) during a period of 14 months (November 2010–December 2011), as part of a larger hospital-based surveillance study designed to understand the etiology and epidemiology of severe pneumonia cases among children. 

## 2. Experimental

This specific analysis is part of a larger study conducted from November 2010 to December 2011 in Rabat, the capital of the kingdom of Morocco, at the *Hôpital d’Enfants de Rabat* (HER), within the infectious and respiratory diseases’ ward. During the 14 month-long study, children aged between 2 and 59 months admitted to HER with respiratory symptomatology were identified by study staff at the pediatric infectious diseases ward and approached for recruitment by trained nurses if they fulfilled WHO’s definition of clinical severe pneumonia (CSP) [28,29], namely: History of cough or reported breathing difficulty and increased respiratory rate (RR) according to age and chest indrawing. Wheezing was not considered an exclusion criterion. Severity was graded using the Respiratory Index of Severity in Children (RISC) score [30].

Children fulfilling inclusion criteria and whose parents had signed an informed consent underwent standardized procedures. Demographic, socio-economic and clinical data (including evolution during admission and outcome) were routinely collected following a standardized questionnaire. Antero-posterior chest X-rays were performed on admission and pulse oximetry (Bionics palm care®) was used to determine oxygen saturation (SaO_2_). Nasal and pharyngeal swabs were performed for diagnosis of bacterial infection/carriage. Venous blood was collected on admission for blood culture, full blood cell count, C-reactive protein (CRP) and procalcitonine (PCT). Treatment for severe pneumonia and other related diagnoses was done according to national guidelines and decided by hospital clinicians. Antibiotic usage during hospitalization was documented and confirmed by study staff, and information regarding antibiotic usage prior to hospitalization relied on parent’s recall.

### 2.1. Laboratory Methods

Samples were analyzed in the medical research laboratory of the *Centre Hospitalier Universitaire Ibn Sina* (CHIS). PCT and CRP levels were determined using miniVIDAS®’ Biomerieux and Microlab300, respectively. Blood samples were cultured using an automated blood culture system (BD Bactec®). Nasopharyngeal samples were cultured in specific media such as Columbia agar, Chocolate agar and Chapman agar. Bacterial isolates obtained from blood and nasopharyngeal swabs were identified by colony morphology and biochemical tests or by using BD-Phoenix® automated system. Antibiotic susceptibility of bacterial isolates was determined in accordance with CA-SFM (*Comité de l’Antibiogramme de la Société Française de Microbiologie*) [31] using BD-Phoenix**®** automated system or by disk diffusion.

### 2.2. Data Management and Statistical Analysis

All study questionnaires were manually verified for completeness and errors, edited whenever necessary and subsequently double entered using a program written in Filemaker Pro 12 (Filemaker Inc., Santa Clara, CA, USA). Statistical analyses were done with Stata 11 (Stata Corp., College Station, TX, USA). Study variables were counted and summarized in frequency tables. Means with corresponding standard deviations, or medians and interquartile ranges are presented, for normally or non-normally distributed variables, respectively. A multivariate logistic regression was performed with antibiotic use/not use as the outcome, using an automated backward and forward stepwise estimation. Variables previously shown in the literature to be related to antibiotic usage and other available data were investigated. All variables that were associated with antibiotic usage at a significance level of *p* < 0.10 in the univariate analysis were included in the initial model. The significance level for removal from the model was set at *p* = 0.06 and that for addition to the model at *p* = 0.05. Strength of association was determined by estimating the odds ratio (OR) and the 95% confidence intervals (CI). 

### 2.3. Ethics

The protocol and other relevant documents of this study (including the informed consent document) were approved by the Ethics Committee of the Hospital Clinic (Barcelona, Spain), and by the Institutional Review Board (*Comité d’Éthique de la recherché Biomédicale*, *Départ* N° 1252-16Déc2009) of the Faculty of Medicine in Rabat (Morocco). All caretakers signed a written informed consent form prior to any study procedure.

## 3. Results and Discussion

### 3.1. Antibiotic Usage Prior to Hospitalization

During the 14-month long study period, 700 children aged ≥2 months and <5 years were recruited to the study. Mean age of recruited patients was 21.5 months (SD 14.6), and 64.1% were male. According to the admission interview to the caretakers, 29.4% (206/700) referred their child receiving antibiotics within the two weeks preceding hospitalization. Antibiotics were principally prescribed by a physician (166/192; 86.5%), or obtained directly at the pharmacy (21/192; 10.9%), and in 5 (2.6%) cases taken as self-medication (usage of personal reserves obtained from relatives/friends). Amoxicillin/clavulanic acid (73/206; 35.4%) and oral amoxicillin (50/206; 24.3%) were the two most frequently used antibiotics at the community level, followed by macrolides (mainly josamycin, 47/206; 22.8%) and cephalosporins (mainly second generation, occasionally third generation; 20/206; 9.7%). In 5 cases (2.4%), patients took cotrimoxazole, and a combination of two antibiotics was used in 11 patients (5.3%). Importantly, no penicillin use was reported.

Table 1 compares the demographic, clinical, laboratory and outcome variables collected among patients recruited to the study according to prior antibiotic usage. Patients having received antibiotics were significantly younger, had a significantly shorter duration of breastfeeding, and a worse compliance with the vaccination schedule in comparison to those not treated. They also showed a longer-lasting and more severe clinical picture (higher mean RISC score [30] and higher feeding difficulties) than those not having received antibiotics. Parents of children not having received antibiotics were more frequently unemployed than those being treated. In terms of evolution post-admission, those children who received antibiotics prior to hospitalization were also significantly more prone to need intra-hospital antibiotics, and showed a worse clinical evolution, including a significantly higher risk of requiring intensive care. Table 2 summarizes those risk factors found to be independently associated with prior antibiotic usage according to the multivariate analysis. Pre-hospitalization treatment with antibiotics was significantly more frequent in those children with symptoms lasting more than seven days (aOR 3.98; 95%CI 2.17–7.31), referring a history of fever (aOR 1.52; 95%CI 1.02–2.28) or anorexia/feeding difficulties (aOR 1.59; 95%CI 1.11–2.28). Households in which there were no smokers (aOR 1.65; 95%CI 1.13–2.40) or where vaccination compliance was not complete (aOR 1.89; 95%CI 1.11–3.23) were also more likely to have received pre-hospitalization antibiotic treatment. 

**Table 1:** Basic demographic, clinical, laboratory and outcome variables among patients recruited to the study according to prior antibiotic usage.

Patient’s variables		Patients with no history of previous antibiotic treatment (N = 494)	Patients having received pre-admission antibiotics (N = 206)	*p* value
Basic demographics	Female gender n/N (%)	173/494 (35.0)	78/206 (37.9)	0.475
	Age in months: Mean (SD)	22.3 (14.8)	19.7 (13.8)	**0.035**
	Infants (<1 year) n/N (%)	140/494 (28.3)	74/206 (35.9)	**0.047**
	Number of siblings: Mean (SD)	2.2 (1.2)	2.4 (1.2)	0.208
Socio-Economic status	At least one parent illiterate n/N (%)	217/494 (43.9)	87/206 (42.2)	0.680
	At least one parent university n/N (%)	60/494 (12.2)	36/206 (17.5)	0.062
	Both parents unemployed n/N (%)	94/494 (19.0)	26/206 (12.6)	**0.040**
	Both parents employed n/N (%)	40/488 (8.2)	18/203 (8.9)	0.772
	Medical insurance n/N (%)	118/490 (24.1)	63/206 (30.6)	0.074
	Proportion of children living in house >6 people n/N (%)	192/494 (38.9)	76/206 (36.9)	0.625
	Number of rooms in house: Mean (SD)	2.2 (1.2)	2.1 (1.1)	0.150
Health history	Prematurity n/N (%)	34/493 (6.9)	18/206 (8.7)	0.398
	No breastfeeding at all n/N (%)	46/493 (9.3)	23/205 (11.2)	0.446
	Breastfeeding >6months n/N (%)	313/492 (63.6)	109/205 (53.2)	**0.010**
	Breastfeeding duration (in months): Mean (SD)	10.1 (7.9)	8.4 (7.5)	**0.008**
	Known asthmatic patient n/N (%)	143/494 (28.9)	48/206 (23.3)	0.126
	Patient with a diagnosed chronic condition n/N (%)	18/493 (3.6)	7/205 (3.4)	0.878
	Prior admission due to respiratory condition n/N (%)	166/494 (33.6)	61/206 (29.6)	0.304
	No smokers at home n/N (%)	284/493 (57.6)	133/206 (64.6)	0.087
Vaccinations and other preventive strategies	Correct vaccines according/age n/N (%)	439/492 (89.2)	167/206 (81.0)	**0.004**
	At least one dose of Hib vaccine n/N (%)	485/494 (98.2)	199/206 (96.6)	0.203
	At least one dose of Pneumococcal vaccine ** n/N (%)	78/494 (15.8)	39/206 (18.9)	0.310
	At least one dose of Vitamine A n/N (%)	455/490 (92.9)	184/206 (89.3)	0.120
Clinical symtomatology and severity on arrival to Hospital	Duration of current symptomatology: Mean (SD)	3.2 (3.9)	5.9 (8.0)	**<0.001**
	History of fever n/N (%)	319/493 (64.7)	156/206 (75.7)	**0.004**
	Axillary temperature (°C): Mean (SD)	37.8 (0.9)	37.8 (0.9)	0.898
	History of feeding difficulties n/N (%)	174/459 (37.9)	92/188 (48.9)	**0.010**
	Respiratory rate: Mean (SD)	58.8 (14.1)	60.6 (14.3)	0.125
	Wheezing n/N (%)	331/494 (67.0)	130/206 (63.1)	0.322
	Crackles n/N (%)	50/494 (10.1)	25/206 (12.1)	0.432
	RISC score: Mean (SD)	1.3 (1.3)	1.5 (1.4)	**0.023**
	Oxyhaemoglobin saturation: Mean (SD)	95.0 (4.0)	94.4 (5.1)	0.110
	Pathological chest X-ray (“Endpoint pneumonia”) n/N (%)	73/414 (17.6)	38/179 (21.2)	0.303
	C-reactive protein CRP (mg/dL): Mean (SD)	2.6 (3.2)	2.9 (3.1)	0.407
	Procalcitonine PCT (ng/mL): Mean (SD)	3.2 (19.8)	3.8 (12.7)	0.692
Evolution during admission	Duration of admission (Days): Median (IQR)	4 (2–7)	5 (2–7)	0.262
	Admissions with duration >10 days n/N (%)	56/494 (11.3)	30/206 (14.6)	0.236
	Admissions requiring oxygen n/N (%)	383/494 (77.5)	152/206 (73.8)	0.288
	Admissions requiring bronchodilators n/N (%)	362/494 (73.3)	139/206 (67.5)	0.121
	Admissions requiring antibiotics n/N (%)	178/494 (36.0)	108/206 (52.4)	**<0.001**
	Admissions requiring ICU n/N (%)	33/493 (6.7)	24/206 (11.6)	**0.029**
	Overall study deaths * n/N (%)	17/486 (3.5)	11/204 (5.4)	0.250

* 10 patients absconded without a definite outcome; ** Conjugate 13-valent Pneumococcal vaccine introduced in Morocco mid-way through the recruitment period.

Pre-admission antibiotic prescription also significantly differed in relation to the final diagnosis given by clinicians at the hospital. Children with bronchitis (74/319; 23.2%) and laryngitis (13/48; 27.1%) received significantly less frequently antibiotics than children with bronchiolitis (40/105; 38.1% or pneumonia of suspected bacterial origin (69/195; 35.4%) (*p* < 0.001).

**Table 2:** Risk factors independently associated with prior to hospitalization antibiotic usage.

Variables (N = 700)	adjusted odds ratio (OR)	95% CI		*p* (LR X^2^ test 1 d.f.)
		Inferior	Superior	
No smokers in the house (n = 417)	1.65	1.13	2.40	0.010
Symptoms lasting >7 days (n = 60)	3.98	2.17	7.31	<0.001
Referred fever history (n = 475)	1.52	1.02	2.28	0.041
Anorexia/Feeding difficulties (n = 266)	1.59	1.11	2.28	0.012
Incorrect vaccination according to age (n = 92)	1.89	1.11	3.23	0.020

### 3.2. Antibiotic Usage during Hospitalization

Of the 700 recruited children, 286 (40.9%) required intra-hospital antibiotherapy. Among patients being prescribed antibiotics, 221 (31.6%) took only one kind throughout hospitalization, while 50 received two (7.2%) and further 15 (2.1%) received three or more. Antibiotics most commonly prescribed included cephalosporins (213/286; 74.5%), macrolides (60/286; 21.0%), gentamicin (39/286; 13.6%) and amoxicillin/clavulanic acid (32/286; 11.2%) or amoxicillin (14/286; 4.9%). The use of chloramphenicol (1 patient), cloxacillin (3 patients) or other antibiotics, including penicillin, was anecdotal among study patients. Most antibiotics were given parenterally (data not shown). 

Table 3 summarizes patient’s characteristics according to antibiotic need during hospitalization. Patients requiring intra-hospital antibiotics were significantly younger, had a longer duration of symptoms prior to hospitalization, and a more severe clinical picture on arrival. Mean axillary temperature and mean RISC score on admission were significantly higher, and patients showed lower mean oxyhaemoglobin saturation, more pathological X-rays with diagnosis of pneumonia and higher mean C-reactive protein or procalcitonin plasma levels. Duration of admission was also significantly prolonged among those receiving antibiotics, and these patients also required more frequently intensive care. Case fatality rates were significantly higher for those patients requiring antibiotics (22/283; 7.8%) than for those not requiring (6/407; 1.5%; *p* < 0.001). On the other hand, patients hospitalized without antibiotics, showed more frequently wheezing, and required significantly more frequently concomitant bronchodilator and oxygen therapy. 

Intra-hospital antibiotic prescription was clearly syndrome-oriented. Ninety-two percent (180/195) of the pneumonia cases of suspected bacterial origin received antibiotics, while only 13.8% (44/319) of the bronchitis episodes were treated with antimicrobials. 

### 3.3. Antibiotic Susceptibility Patterns of Circulating Respiratory Bacteria

As previously reported [32] the yield of blood culture positivity within this surveillance project was low, and only 14/689 (2.0%) of the patients had a confirmed bacteremia, two of which caused by *S. pneumonia*, one b*y Hib*, and the remaining 11 caused by various gram negative microorganisms. Susceptibility patterns to these microorganisms (data not shown) were very variable. 

**Table 3:** Demographic, clinical, laboratory and outcome variables among patients recruited to the study according to antibiotic usage during hospitalization.

Patient’s variables		Patients not given antibiotics during admission	Patients requiring antibiotics during admission	*p* value
		n/N (%)	n/N (%)	
Basic demographics	Female gender	138/414 (33.3)	113/286 (39.5)	0.094
	Age in months: Mean (SD)	25.0 (15.0)	16.4 (12.2)	<0.001
	Infants (<1 year)	91/414 (22.0)	123/286 (43.0)	<0.001
Clinical symtomatology and severity on arrival to Hospital	Duration of current symptomatology: Mean (SD)	3.0 (4.3)	5.3 (6.7)	<0.001
	History of fever	228/414 (55.1)	247/285 (86.7)	<0.001
	Axillary temperature (°C): Mean (SD)	37.5 (0.7)	38.2 (0.9)	<0.001
	History of feeding difficulties	146/383 (38.1)	120/264 (45.5)	0.062
	Respiratory rate: Mean (SD)	57.7 (12.6)	61.6 (16.0)	<0.001
	Wheezing	351/414 (84.8)	110/286 (38.5)	<0.001
	Crackles	28/414 (6.8)	47/286 (16.4)	<0.001
	Oxyhaemoglobin saturation: Mean (SD)	95.4 (3.0)	93.9 (5.6)	<0.001
	Pathological chest X-ray (“Endpoint pneumonia”)	21/351 (6.0)	90/242 (37.2)	<0.001
	C-reactive protein CRP (mg/dL): Mean (SD)	1.7 (2.2)	4.1 (4.2)	<0.001
	Procalcitonine PCT (ng/mL): Mean (SD)	0.6 (3.5)	7.3 (27.3)	<0.001
Evolution during admission	Duration of admission (Days): Median (IQR)	4 (2-6)	5 (3-8)	0.001
	Admissions with duration >10 days	31/414 (7.5)	55/286 (19.2)	<0.001
	Admissions requiring oxygen	370/414 (89.4)	165/286 (57.7)	<0.001
	Admissions requiring bronchodilators	379/414 (91.6)	122/286 (42.7)	<0.001
	Admissions requiring ICU	8/413 (1.9)	49/286 (17.1)	<0.001
	RISC score: Mean (SD)	0.8 (1.0)	2.1 (1.4)	<0.001
	Overall study deaths *	6/407 (1.5)	22/283 (7.8)	<0.001

* 10 patients absconded without a definite outcome.

In order to further understand the susceptibility patterns to commonly used antibiotics in the area, respiratory bacteria obtained from the patient’s nasopharynx [*Streptococcus pneumoniae* (n = 155; 22.1% of all patients); *Haemophilus influenzae* type b (n = 84; 12.0%); *Staphylococcus aureus* (n = 44; 6.3%) and *Moraxella catharralis* (n = 200; 28.6%)] were studied. Antibiotics tested and susceptibility profiles can be seen in Table 4. *S. pneumoniae* generally showed good susceptibility profiles to most antibiotics tested, and documented resistance was only seen in 20/98 (20.4%) isolates to erythromycin, and in 38/154 (24.7%) isolates to Trimethoprim/sulfamethoxazole (cotrimoxazole). Resistance of *S. pneumoniae* to Penicillin G and amoxicillin was 10.3% and 14.6%, respectively. *Haemophilus influenzae* type b (Hib) strains showed similar moderate resistance to amoxicillin/clavulanic acid (23/83; 30.1%), amoxicillin (27/83; 32.5%) or ampicillin (27/84; 32.1%), but were highly susceptible to cephalosporins, chloramphenicol or gentamicin (susceptibility above 95% in all cases). Fifty nine percent (47/80) of Hib isolates were resistant to cotrimoxazole. Finally, *S. aureus* and *M. catharralis* isolates showed high levels of resistance to penicillin, ampicillin or amoxicillin (>92% resistance), but remained highly susceptible to amoxicillin/clavulanic acid or chloramphenicol (90% and 99.5% respectively). 

**Table 4:** Antibiotic susceptibility patterns of respiratory bacteria detected in nasopharynx of hospitalized children with clinical severe pneumonia.

Bacteria	Antibiotics											
		Amoxicillin-Clavulanic	Amoxicillin	Ampicillin	Cefotaxime	Ceftriaxone	Chloramphenicol	Ciprofloxacin	Erythromycin	Penicillin G	Gentamicin	Cotrimoxazole
		n/N (%)	n/N (%)	n/N (%)	n/N (%)	n/N (%)	n/N (%)	n/N (%)	n/N (%)	n/N (%)	n/N (%)	n/N (%)
*Strept. pneumoniae*	*S*	100/100 (100)	31/55 (56.4)	100/100 (100)	139/155 (89.7)	100/100 (100)	148/154 (96.1)	100/100 (100)	78/98 (79.6)	108/155 (69.7)	-	106/154 (68.8)
*(n = 155)*	*I*	-	16/55 (29)	-	15/155 (9.7)	-	-	-	-	31/155 (20.0)	-	10/154 (6.5)
	*R*	-	8/55 (14.6)	-	1/155 (0.7)	-	6/154 (3.9)	-	20/98 (20.4)	16/155 (10.3)	-	38/154 (24.7)
*Haemophilus influenzae b*	*S*	58/83 (69.9)	56/83 (67.5)	57/84 (67.9)	83/84 (98.8)	83/84 (98.8)	81/83 (97.6)	83/84 (98.8)	-	-	79/83 (95.1)	32/80 (40)
*(n = 84)*	*I*	-	-	-	-	-	-	-	-	-	-	1/80 (1.2)
	*R*	23/83 (30.1)	27/83 (32.5)	27/84 (32.1)	1/84 (1.2)	1/84 (1.2)	2/83 (2.4)	1/84 (1.2)	-	-	4/83 (4.9)	47/80 (58.8)
*Staph. aureus*	*S*	27/30 (90)	2/43 (4.6)	2/43 (4.6)	40/44 (90.9)	40/44 (90.9)	28/29 (96.6)	26/29 (89.7)	9/10 (90)	2/43 (4.6)	39/43 (95.4)	42/44 (95.4)
*(n = 44)*	*I*	-	-	-	-	-	-	-	-	-	-	-
	*R*	3/30 (10)	41/43 (95.4)	41/43 (95.4)	4/44 (9.1)	4/44 (9.1)	1/29 (3.4)	3/29 (10.3)	1/10 (10)	41/43 (95.4)	2/43 (4.6)	2/44 (4.6)
*Moraxella catharralis*	*S*	198/199 (99.5)	16/200 (8.1)	16/200 (8.1)	-	-	198/198 (100)	190/190 (100)	-	-	190/190 (100)	169/198 (85.3)
*(n = 200)*	*I*				-	-	-	-	-	-	-	14/198 (7.1)
	*R*	1/199 (0.5)	184/200 (92)	184/200 (92)	-.	-	-	-	-	-	-	15/198 (7.6)

S = Susceptible; I = Intermediate; R = Resistant.

### 3.4. Discussion

Data on antibiotic prescription and usage in the community and within hospitals within a specific setting can be helpful to inform policy makers on the adequacy of their recommendations for the treatment of specific infections, particularly if they can be linked to epidemiological surveillance of most frequent etiologies. In Morocco, the burden of disease secondary to pediatric ARI remains unacceptably high, with many infections requiring hospital admission or even critical care, and with a high associated case fatality rate (4.1% among our recruited patients). Thus, the use of antibiotics has become understandably a critical element in currently existing programs to manage respiratory infections; both at the community level and once patients have been hospitalized. In our population, 30% of the children admitted for severe ARI had received antibiotics before their admission. This low-to-moderate figure is lower to that reported at the national level for outpatient treatment of ARIs [23], or to that reported in other settings [33,34,35]. Indeed, prescription of antibiotics for ARIs at the outpatient level tends to be high, despite the fact that the majority of respiratory infections will progress adequately irrespective of treatment. An important limitation of our study derives from only including admitted patients (*i.e.*, those patients more severe or not responding to previous treatment, or which go to hospital because they haven’t previously received treatment), a population that surely differs from the overall outpatient community, which is likely prescribed more often antibiotics than in this series [33]. Moreover, this series obtained in a referral tertiary level hospital located within an urban setting may also not be fully representative of what is occurring in other more rural areas in Morocco. However, whenever assessing antimicrobial prescription attitudes at the community level, the type of antibiotics prescribed matter more than the absolute figure of children receiving drugs. In this respect, the high extra-hospital use of extended spectrum antibiotics (amoxicillin-clavulanic acid; macrolides, cephalosporins, *etc*.) and virtually inexistent utilization in our series of penicillin, a drug that remains first line recommendation for the treatment of most community-acquired upper respiratory tract infections in many developed countries [36], raises some concerns, although could be related to the fact that most episodes among this series were of lower respiratory tract origin. Indeed, national Moroccan recommendations still include the use of penicillin for acute tonsillitis and certain other upper respiratory tract infections [37], although its use at the community level for those indications has been progressively replaced by other antibiotics such as oral amoxicillin or macrolides. At the hospital, however, the lack of widespread availability of timely microbiology results and antibiotic susceptibility patterns for detected isolates hinders the use of narrow-spectrum antibiotics such as penicillin even for infections that could be fully susceptible to this antimicrobial. 

Independent risk factors associated with pre-hospitalization usage of antibiotics in Rabat seem mostly related to the severity of the clinical picture or the duration of the illness. Indeed, symptoms lasting over a week, or the presence of fever or feeding difficulties, were all independently associated with a significantly higher antibiotic usage, as previously described in the literature [34]. Young age did not appear as an independent risk factor for antibiotic usage, although children receiving pre-admission antibiotics were significantly younger than those not receiving them. Although other series in developed countries have shown inverse trends [36], with more antibiotics prescribed to older children than to younger ones, our data indicate that in Morocco physicians are less conservative when treating infants. An incorrect vaccination status was also associated with a higher risk of having received antibiotics prior to hospitalization, perhaps in relation to a poorer protection against the vaccine-preventable pathogens associated with ARIs, or as a result of the perception of extra-hospital physicians of this risk. Finally, and more difficult to interpret, was the finding that antibiotic usage was more frequent in households where there were no smokers. We cannot offer with our data a reasonable explanation for this finding, which merits further research, but we can hypothesize that children in households were there are smokers may show a tendency to have more bronchospasm, whereas children in houses without smoking may only come to hospital because of true infections.

During hospitalization, prescription of antibiotics seems also to be syndrome-based, and judicious. Surprisingly, and despite all of the recruited patients showing symptoms of severity, the use of antibiotics was not indiscriminate and was based on a good syndromic orientation, being more frequent in those cases oriented towards a bacterial origin, and much lesser among the wheezers. Not surprisingly, cases with greater severity on admission also were more prone to receive antibiotics. The establishment of protocols at the HER concerning antibiotic prescription and the choice of antimicrobials for ARIs precedes existing guidelines proposed by the National pediatric societies [37], and are based on currently existing and well-established international recommendations, which when correctly followed at the ward seem to have helped to minimize antibiotic misuse in other settings [38]. 

Susceptibility from common circulating respiratory bacteria to commonly used antibiotics remains reasonably high, a finding that differs from previous reports in Moroccan ARI isolates [24,25]. A major limitation of this study is that the incidence of invasive bacterial disease detected among recruited patients was low (24/690; 3.5%) and of multiple etiologies, thus not allowing us to investigate susceptibility patterns among invasive isolates. However, we have presented antibiotic susceptibility patterns among respiratory bacteria carried in the nasopharynx. It is clear that susceptibility patterns among invasive isolates and non-invasive commensal bacteria in the upper respiratory tract may be very different [39,40], but it is however reassuring to find that circulating bacteria remain highly sensitive to commonly used antibiotics. 

The aforementioned high extra-hospital use of antibiotics such as macrolides or amoxicilllin plus clavulanic acid may favor the selection of antibiotic resistant pathogenic microorganisms. Thus, despite the low levels of antimicrobial resistance detected in the series, it is of concern the presence of >20% of *S. pneumoniae* exhibiting erythromycin resistance. As the most frequent mechanisms of macrolides resistance in Gram-positive microorganisms are encoding in transferable elements [41] usually together with other antibiotic resistance determinants, the possibility of a long dissemination of these elements is a worrying scenario.

Besides the aforementioned limitations, this study has other limitations worth mentioning, including the fact that prior antibiotic use at the community level was only recorded as per caretaker’s report, and was not confirmed y other means. Also, while we were able to describe antibiotic usage as a binary variable, this study could not describe in detail factors such as the duration of treatment, doses and administration schemes, or changes in the antibiotics utilized (and rational for that). Such information appears relevant to further understand the perceptions and attitudes of both antibiotic prescribers and users, and also to shed a light on selection pressure on pathogens. It may also be useful to orient on further needs for continuous education of physicians in clinical pharmacology [42]. 

## 4. Conclusions

Pre-admission antibiotic prescription patterns among this series of Moroccan children with respiratory conditions appear conservative, and were associated with severity and duration of the underlying respiratory disease. Antibiotic usage during hospitalization seems also to be judicious and well delimited for cases of suspected bacterial etiology, or whenever a life-threatening condition is present on arrival. This rational use of antibiotics, occurring in a context where there is no control over the prescription and sale of antibiotics, and where access to drugs is widespread, needs to be praised. Antibiotic susceptibility to commonly used antibiotics of circulating respiratory bacteria usually carried in pediatric patient’s nasopharynx remains reasonably high, but needs regular monitoring, particularly among invasive isolates, to detect warning signs of emerging antibiotic resistance.
